# Discriminative compensatory activation during auditory beat perception in Parkinson’s disease and multiple system atrophy

**DOI:** 10.3389/fnins.2026.1700800

**Published:** 2026-01-30

**Authors:** Chung-Yao Chien, Chi-Lun Lin, Jack Shen-Kuen Chang, Tsung-Lin Lee, Tien-Yu Lin, Chou-Ching Lin

**Affiliations:** 1 Department of Neurology, National Cheng Kung University Hospital, College of Medicine, National Cheng Kung University, Tainan, Taiwan; 2 Department of Biomedical Engineering, National Cheng Kung University, Tainan, Taiwan; 3 Department of Mechanical Engineering, National Cheng Kung University, Tainan, Taiwan; 4 Medical Device Innovation Center, National Cheng Kung University, Tainan, Taiwan; 5 Cross College Elite Program, National Cheng Kung University, Tainan, Taiwan

**Keywords:** beat perception, fMRI, multiple system atrophy, Parkinson’s disease, rhythmic auditory stimulation

## Abstract

**Introduction:**

Auditory beat perception (ABP) is crucial for rhythmic auditory stimulation (RAS), a therapeutic approach for neurological disorders, and particularly Parkinson’s disease (PD). The mechanism is thought to be associated with increased activation of the cerebellum and motor-related regions. However, few studies have explored its effects on patients with multiple system atrophy (MSA). This study aimed to compare the compensatory mechanism of ABP in patients with PD and MSA.

**Methods:**

Participants with PD and MSA and normal controls (NCs) were recruited from National Cheng Kung University Hospital. During task-based functional MRI (fMRI), the participants listened to rhythmic beat sounds using a boxcar paradigm. Basic characteristics and brain volumes were compared between the PD and MSA groups, and fMRI findings were further compared with the NCs.

**Results:**

There were no significant differences in age, gender, motor scores or disease duration between PD (*n* = 16) and MSA (*n* = 14) groups. Brain volume analysis revealed a significant reduction in cerebellar volume in the MSA group. ABP analysis via fMRI demonstrated greater activation in the left premotor cortex in MSA groups compared to NCs. Activation in the right cerebellum was significantly higher in PD group but absent in the MSA group. Different correlation trends between activation and cerebellar volume were observed.

**Conclusion:**

ABP in the PD and MSA groups showed distinct compensatory mechanisms by cerebellar and premotor cortex activation, respectively. These findings provide insights into compensatory mechanisms regarding RAS therapy in patients with atypical parkinsonian disorders.

## 1 Introduction

Rhythmic auditory stimulation (RAS) is used to treat many neurological disorders, including Parkinson’s disease (PD) (Ghai et al., 2018; Proksch et al., 2020; Kasdan et al., 2022; Emmery et al., 2023; Koshimori and Thaut, 2023; Ashoori et al., 2015), Alzheimer’s dementia (Wang et al., 2024) and stroke (Ghai and Ghai, 2019; Gonzalez-Hoelling et al., 2024; Wang et al., 2022). The first step in RAS is beat perception through the activation of not only the auditory cortex but also motor-related areas including premotor and sensorimotor regions, basal ganglia and cerebellum (Damm et al., 2020). Beat perception is then followed by entrainment of neuronal activity oscillations along with the given external tempo measured by electroencephalography or magnetoencephalography (Fujioka et al., 2012). The final step is to synchronize motor output with the entrained neuronal activities, termed synchronization (Nozaradan et al., 2015). Through this reciprocal and feedback process, motor output can be adjusted by a pattern of neuronal activity changes during auditory rhythm stimulation. Under normal conditions, beat perception creates multiple motor, sensory and cerebellar activations simultaneously, and this mechanism may explain how RAS subconsciously improves movement, including gait. Many studies have investigated beat perception and brain functional alterations in patients with PD, and shown greater activation than in normal controls. This phenomenon is thought to be a compensatory response in order to accomplish motor output by following entrainment and synchronization.

Multiple system atrophy (MSA) is an atypical parkinsonian disorder, in which the associated neurodegeneration causes parkinsonian and cerebellar symptoms. Due to the distinctive pathological changes and disease progression, conventional pharmacological therapies such as dopaminergic agents usually fail to elicit significant improvements in gait or mobility. However, it is unclear whether the prominent clinical effects RAS has shown in patients with PD are also applicable to patients with MSA. To date, few studies have investigated the effect of RAS on atypical parkinsonian disorders, and only one pilot study (Pantelyat et al., 2022) has shown a positive effect of RAS on patients with MSA (*n* = 8). In that study, RAS improved gait velocity and step length, but did not affect cadence. However, the effect of RAS on a larger group of patients with MSA and the overall responses are unknown due to the multiple system involvement in brain networks and structural differences in MSA. Gait impairment in parkinsonian disorders is closely linked to balance dysfunction (Lena et al., 2023), and the prominent cerebellar impairment in MSA likely imposes greater limitations on the effectiveness of gait training with RAS.

Previous studies have demonstrated differences between PD and MSA through neuroimaging. One voxel-based morphometry study revealed reduced volumes in the basal ganglia, middle and inferior cerebellar peduncles, pons, and throughout the cerebrum in patients with MSA compared to those with PD (Planetta et al., 2015), as also reflected by microstructural changes in diffusion imaging. MSA is associated with greater changes in diffusivity in the putamen, cerebellar peduncle and corticospinal tract compared to PD (Pasquini et al., 2022). However, PD was associated with more extensive activation deficits throughout the cerebrum compared to MSA in a functional MRI (fMRI) study of patients performing a hand grasping motor task (Planetta et al., 2015). Subtle functional differences between PD and MSA in the bilateral motor cortex (M1) have also been demonstrated in patients undergoing task-based fMRI combined with movements elicited by magnetic transcranial stimulation. These findings suggest that both disorders, although both involving motor deficits, may exhibit distinct patterns of motor area activation (Li et al., 2024). A previous study showed greater hypoperfusion in the putamen in patients with MSA compared to those with PD in perfusion SPECT imaging (Saeed et al., 2020). In addition, a larger scale analysis comparing PD and MSA reported hyperconnectivity in the cerebellar network and hypoconnectivity between cerebellar and frontoparietal networks in patients with PD. Moreover, hypoconnectivity between cerebellar and ventral attention networks was also shown in the MSA group (Tang et al., 2022). These differences in functional neuroimaging findings between PD and MSA may also explain the different responses to RAS.

In this study, we compared auditory beat perception (ABP) between patients with PD and MSA using fMRI. The aim of the study was to evaluate the potential therapeutic use of RAS in patients with MSA.

## 2 Methods

### 2.1 Participants

Patients with PD and MSA who presented with parkinsonism and were older than 30 years were enrolled from movement disorder clinics at National Cheng Kung University Hospital (NCKUH). PD was diagnosed according to the MDS-clinical diagnostic criteria (Postuma et al., 2015) as clinically probable PD or clinically established PD. MSA was diagnosed based on the MDS-MSA diagnostic criteria (Wenning et al., 2022) as clinically probable MSA, clinically probable MSA or clinically established MSA. The Unified Parkinson’s Disease Rating Scale (UPDRS) part III and Montreal Cognitive Assessment (MoCA) test were conducted by a well-trained study nurse. Patients whose routine clinical brain MRI showed prominent white matter changes, ventricular dilatation, or small vessel diseases were excluded. To compare fMRI findings with the PD and MSA groups, a normal control (NC) group of volunteers from NCKUH who were younger and healthy without neurodegenerative disorders were also included. Although the NC group is younger, and analysis may therefore include age-related effects, the primary purpose of the comparison is to demonstrate differences between PD and MSA.

Ethics approval (No. B-ER-112-076) for the study was obtained from the NCKUH Institutional Review Board research ethics committees. The participants were informed of the objectives and procedures of this study, and all signed a written consent form.

### 2.2 Procedures

**Figure 1:**
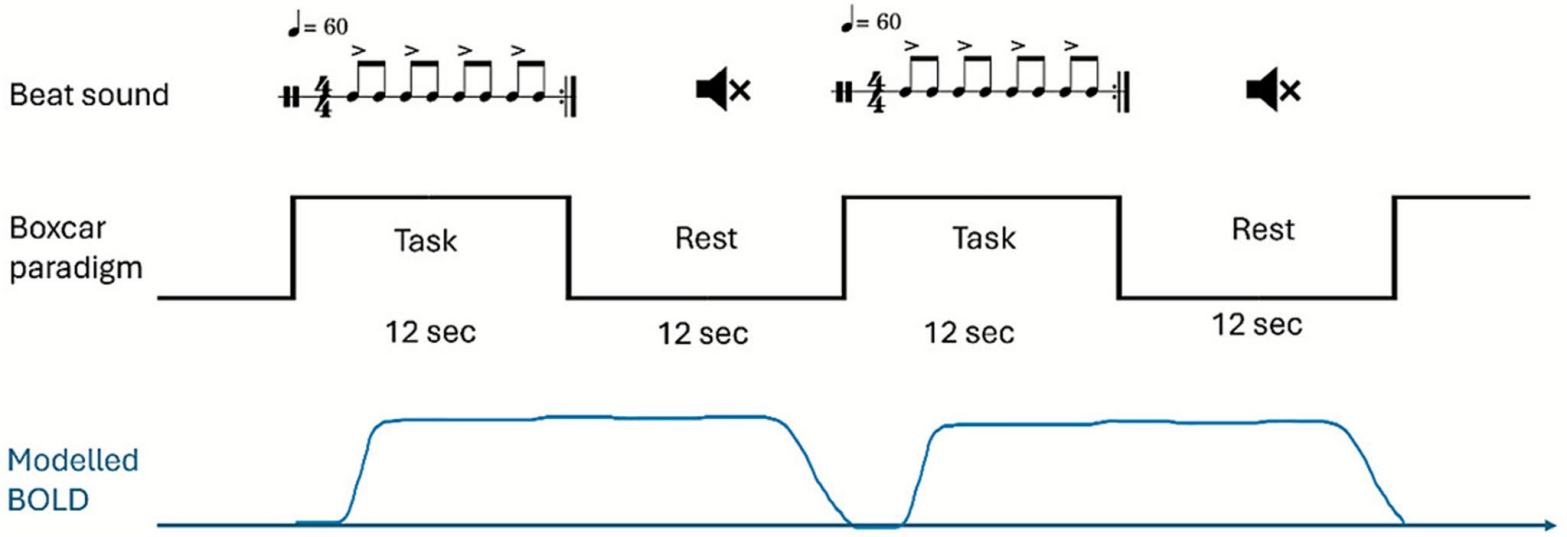
Experimental paradigm of fMRI. In this boxcar paradigm design, the beat sounds were simulated by MIDI, given with a tempo of 60 beats per minute. The eighth notes were accented alternatively to create a more prominent rhythm as usually heard in music. Each task contained three metrics of beat with a duration of 12 s. The rest period was silence without any beat sound. The change in simulated BOLD signal after initiation of the sound stimuli was convolved with the hemodynamic response function. The simulated BOLD signal increased after the task began, peaked at 4 s, and then maintained this level until returning to baseline at about 10 s after the task had stopped (1–2 s before the next auditory stimuli train), as shown in the lower part of the figure (blue line).

The auditory beat was given as the sound of a drum produced using a Musical Instrument Digital Interface (MIDI). The tempo of the beat sound was 120 beats per minute, but alternative notes were accented to elicit greater responses of beat perception (Grahn and Rowe, 2009). The overall rhythmic perception was 60 beats per minute (Figure 1). The participants were asked to listen to the beat sounds attentively. To confirm that the participants could hear the cues and rhythmic sound properly, we first confirmed that they could hear the sound of cues in the MRI chamber during a dummy scan before the actual fMRI scan. Moreover, during the actual measurement, the sound was continually monitored by the examiner. The fMRI procedure followed a boxcar paradigm, and the duration of each task or rest period was 12 s. Thus, the task period contained 12 accented ~100 ms beat sounds interleaved with 12 non-accented ~50 ms beat sounds. The rest period was silence without any auditory stimuli. The simulated blood oxygen level dependent (BOLD) signal increased to a peak of about 4–5 s after the auditory stimuli had been initiated, and returned to baseline at about 10 s during the following rest period (1–2 s before the next auditory stimuli train) according to hemodynamic response function analysis convolved with 12 s of stimulation (Figure 1).

### 2.3 MRI acquisition and analysis

MRI acquisition was performed using a Discovery MR750 3.0 T MR system (GE HealthCare, Chicago, IL, USA). The image analysis included structural T1-weighted MRI and fMRI. The voxel size of T1-weighted MRI was set to 1 mm with the fast spoiled gradient-echo (FSPGR) method and 176 slices. For fMRI, the echo-planar imaging technique was used with an acquisition time to repeat of 2.0 s (300 repeats). The fMRI acquisition time was 10 min, and the field of view was 224 mm with a matrix size of 64 × 64 and 40 slices covering the whole brain, including the cerebellum.

All images were processed using the Statistical Parametric Mapping toolbox (SPM12, Wellcome Trust Center for Neuroimaging, London, UK). The T1-weighted images were obtained for both co-registration of functional images and segmentation of gray matter and computation of regional volumes using the computational anatomical toolbox (CAT12). All toolboxes were implemented using MATLAB (R2020b, MathWorks, Natick, MA, USA). Functional images were preprocessed using components of the CONN toolbox pipeline, including motion correction and denoising to remove outliers, and were subsequently processed with SPM12. The smoothing window in the preprocess was set as 8 × 8 × 8 mm.

### 2.4 The relationship between gray matter volume and activation by auditory beat

After structural MRI and fMRI analyses, the relationships between gray matter volume, and especially the cerebellum, with activation levels were investigated. For fMRI, BOLD signal analysis compared voxel-wise activation across both the entire brain and localized regions. Activation indices for ABP were defined as beta values at specific voxels based on group comparison results. The beta value, or parameter estimate, is the regression coefficient derived from the general linear model in task-based fMRI. It reflects the degree of fitness between the observed BOLD signal and the modeled hemodynamic response to stimuli.

### 2.5 Statistics

Most comparisons were performed between the PD and MSA groups. The NCs were recruited to serve as a comparison group in the fMRI analysis. Gender distribution was evaluated using Fisher’s exact test, with a two-tailed *p* value < 0.05 being considered statistically significant. Age, UPDRS-III score, MoCA score and structural MRI volume of the gray matter, striatum and cerebellums were compared between the PD and MSA groups using two-sample *t*-tests. In addition, fMRI analysis was performed using SPM12 to evaluate differences between the PD and MSA groups (2nd level analysis). Statistical significance was assessed using cluster-level family wise error (FWE) correction, *p* < 0.05. In correlation analysis, normality was checked using the Shapiro–Wilk test (*α* = 0.05). Correlations were tested using Pearson’s correlation coefficients or Spearman rank correlation coefficients.

## 3 Results

### 3.1 Participants’ characteristics

Thirty parkinsonian patients (16 with PD and 14 with MSA) were enrolled (Table 1). In PD group, 10 participants were clinically probable PD, whereas 6 were clinically established PD. The mean ages of the PD and MSA patients were 56.75 and 58.14 years, respectively, and the difference was not statistically significant. The disease duration was relatively longer in the PD group (mean 4.44 years) than in the MSA group (mean 3.71 years), but the difference did not reach significance. Although there was a significant difference in median Hoehn-&-Yahr (HY) stage between the PD and MSA groups (2 vs. 3, *p* < 0.001), the difference in UPDRS-III score was not significant (21 vs. 24.71, *p* = 0.280). Other characteristics including gender and MoCA score were similar in both groups. Regarding laterality (most affected side) in the PD group, 10 participants exhibited right-sided predominance and 6 exhibited left-sided predominance. In MSA group, 8 were clinically probable MSA and 6 were clinically established MSA, 10 participants were cerebellar subtype (MSA-C), whereas 4 were parkinsonian subtype (MSA-P). Fourteen healthy NCs were also enrolled, and they were significantly younger (mean age 33.57 years).

**Table 1:** Demographic and clinical characteristics.

Characteristics	PD (*n* = 16)	MSA (*n* = 14)	NC (*n* = 14)	*p*-value (PD vs. MSA)
Gender (M/F)	10/6	6/8	8/6	0.464
Age (years old)	56.75 (8.68)	58.14 (9.46)	33.57 (7.09)	0.780
Disease duration (years) (range)	4.44 (3–7)	3.71 (3–5)	N/A	0.061
Median HY stage (range)	2 (1–3)	3 (2–3)	N/A	<0.001
UPDRS-III	21 (5.82)	24.71 (10.27)	N/A	0.280
MoCA	26.63 (2.60)	25.07 (2.53)	N/A	0.110

### 3.2 Total brain gray matter volume, cerebellar and striatal volume in each group

**Figure 2:**
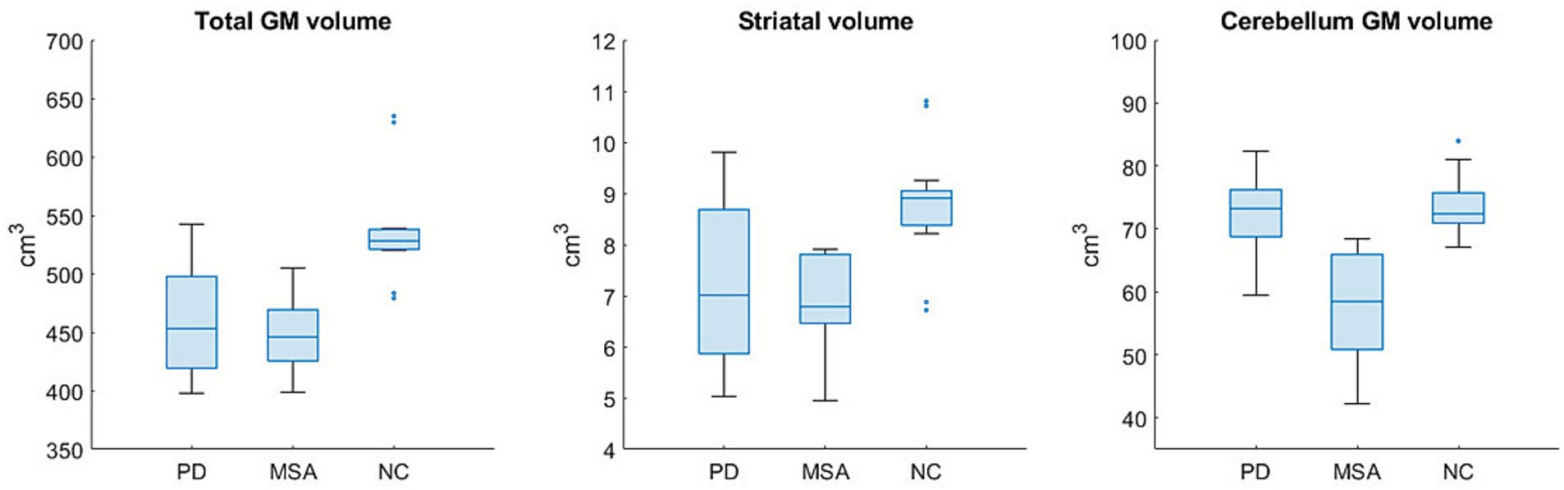
The total gray matter (GM) volume of the brain, striatal, and cerebellar volumes in the three groups. The absolute volume of the cerebellum was smaller in the MSA group than in the other two groups. The NC group had significantly larger volumes of whole brain GM and striatum than the other two groups.

The mean total gray matter volume was 459.82 ± 48.68 cm^3^ in the PD group and 448.20 ± 32.92 cm^3^ in the MSA group, and the difference was not significant. In addition, the mean striatal volume was 7.25 ± 1.65 cm^3^ in the PD group and 6.75 ± 1.26 cm^3^ in the MSA group, also with no significant difference. With respect to laterality in PD group, the mean striatal volume of the most affected side was 7.22 ± 1.65 cm^3^, whereas that of the less affected side was 7.28 ± 1.76 cm^3^; this difference was not statistically significant. For the cerebellum, there was a significantly smaller volume in the MSA group compared to the PD group (57.31 ± 9.38 cm^3^ vs. 72.35 ± 6.24 cm^3^; *p* = 0.0005). In the NC group, the mean total gray matter volume was 536.25 ± 44.40 cm^3^, which was significantly larger than that in both the PD and MSA groups. In addition, the striatal volume in the NC group was 8.81 ± 1.13 cm^3^, which was significantly larger than that in the MSA group (*p* = 0.0001) and that in the PD group (*p* = 0.0059). The cerebellar volume in the NC group was 73.36 ± 4.93 cm^3^, which was slightly larger than that in the PD group, although the difference was not significant (*p* = 0.631). Detailed comparisons of basic characteristics and volumes among the three groups are illustrated in Figure 2 and shown in the Supplementary Table.

### 3.3 Differences in activation by ABP between groups

**Figure 3:**
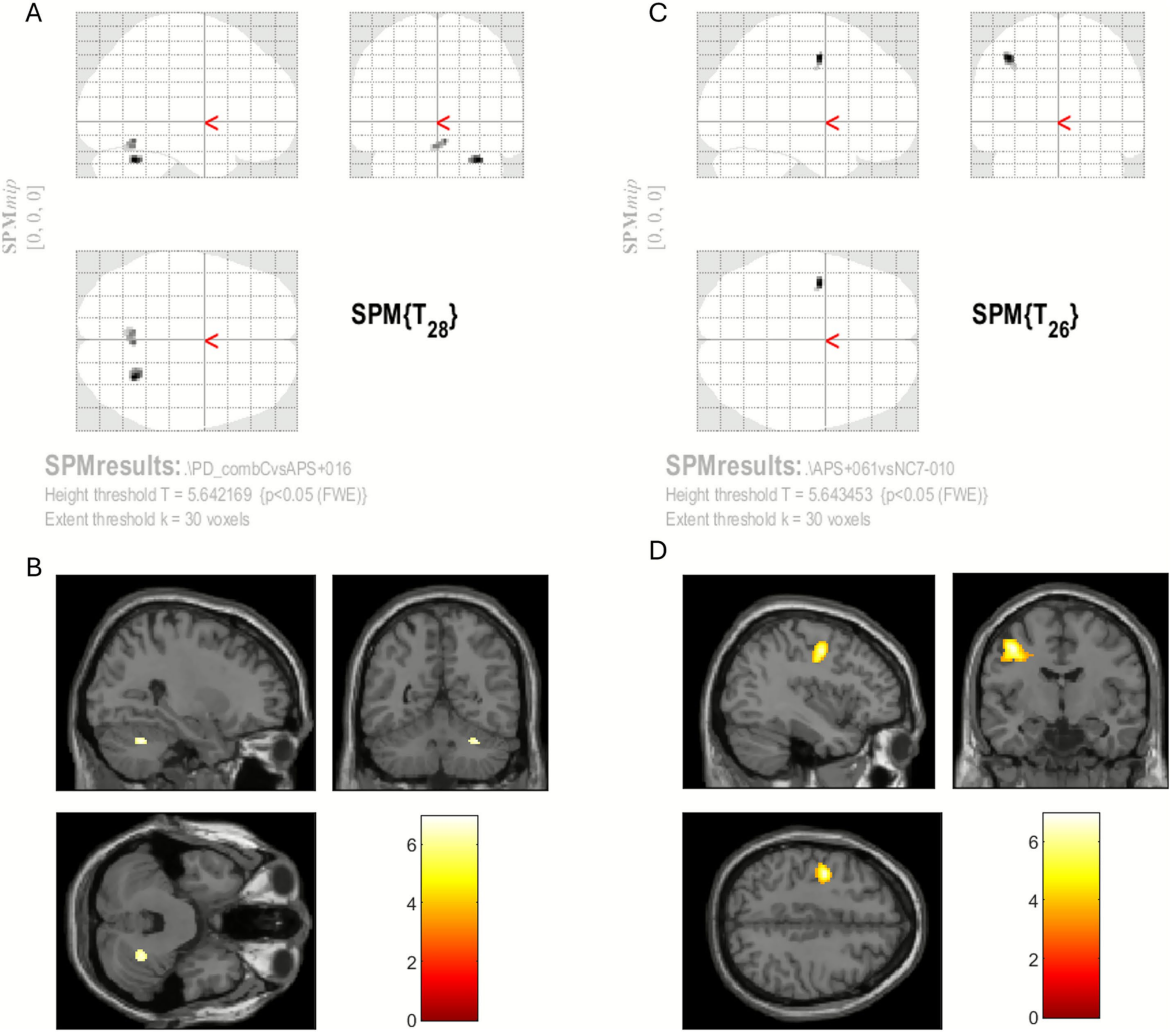
The activation differences between groups. **(A,C)** The raw activation maps in SPM. The distribution of significant differences can be seen globally. **(B,D)** Comparisons between the groups mapping the results onto T1 structural images. **(A,B)** Contrast between PD and MSA (PD > MSA), the main suprathreshold cluster was in the cerebellum (Cerebelum_6_R, *x* = 30, *y* = −58, *z* = −30). **(C,D)** Contrast between MSA and NC (MSA > NC), the main cluster was in the precentral area of the left frontal lobe (*x* = −40, *y* = −6, *z* = −46). The results are illustrated by a significance level of cluster-level FWE correction with *p* < 0.05 and a cluster size > 30 voxels. The color bars are the *T* scores.

Differences in activation between the PD vs. MSA and MSA vs. NC groups are illustrated in Figures 3A–D. Under the setting of cluster-level FWE correction (*p* < 0.05) with a minimum cluster size of > 30 voxels to avoid potential artifacts of edge effect, the contrast between PD and NC (PD > NC) showed no suprathreshold cluster. The contrast between MSA and NC (MSA > NC) showed a clusters of activation in the left precentral region (premotor cortex) [−40, −6, 46]. In the contrast between PD and MSA (PD > MSA), the greatest difference was in the right lateral cerebellum [30, −58, −30] and right intermediate cerebellar region [4, −58, −16] near the vermis. Details of cluster sizes and locations are listed in Table 2. Other contrast condition: NC > PD, NC > MSA and MSA > PD showed no suprathreshold cluster. To further explore potential effects, a more lenient threshold using an uncorrected *p* value < 0.001 with a cluster size of 10 was also applied. Although this approach yielded more informative findings, it carried an increased risk of false positives. Nevertheless, the overall compensatory trends were largely consistent. PD showed a tendency toward increased cerebellar activation, whereas MSA tended to exhibit activation in motor-associated regions without concomitant cerebellar involvement (see Supplementary materials).

**Table 2:** Comparison of activation when auditory beat perception between groups.

Contrast	Region (aal^a^)	p(FWE^b^)	cluster size (K_E_)	*x* (mm)	*y* (mm)	*z* (mm)
PD > MSA	Cerebelum_6_R^c^	0.001	50	30	−58	−30
	Intermediate cerebellum_R	0.002	36	4	−58	−16
MSA > NC	Precentral_L	0.004	34	−40	−6	46
PD > NC	No suprathreshold cluster	N/A	N/A	N/A	N/A	N/A

a aal, automated anatomical labelling atlas.

b Cluster-level family wise error (FEW) correction.

c Cerebelum_6_R: Right middle lobule of lateral hemisphere.

### 3.4 The relationship between cerebellar volume and activation by ABP

**Figure 4:**
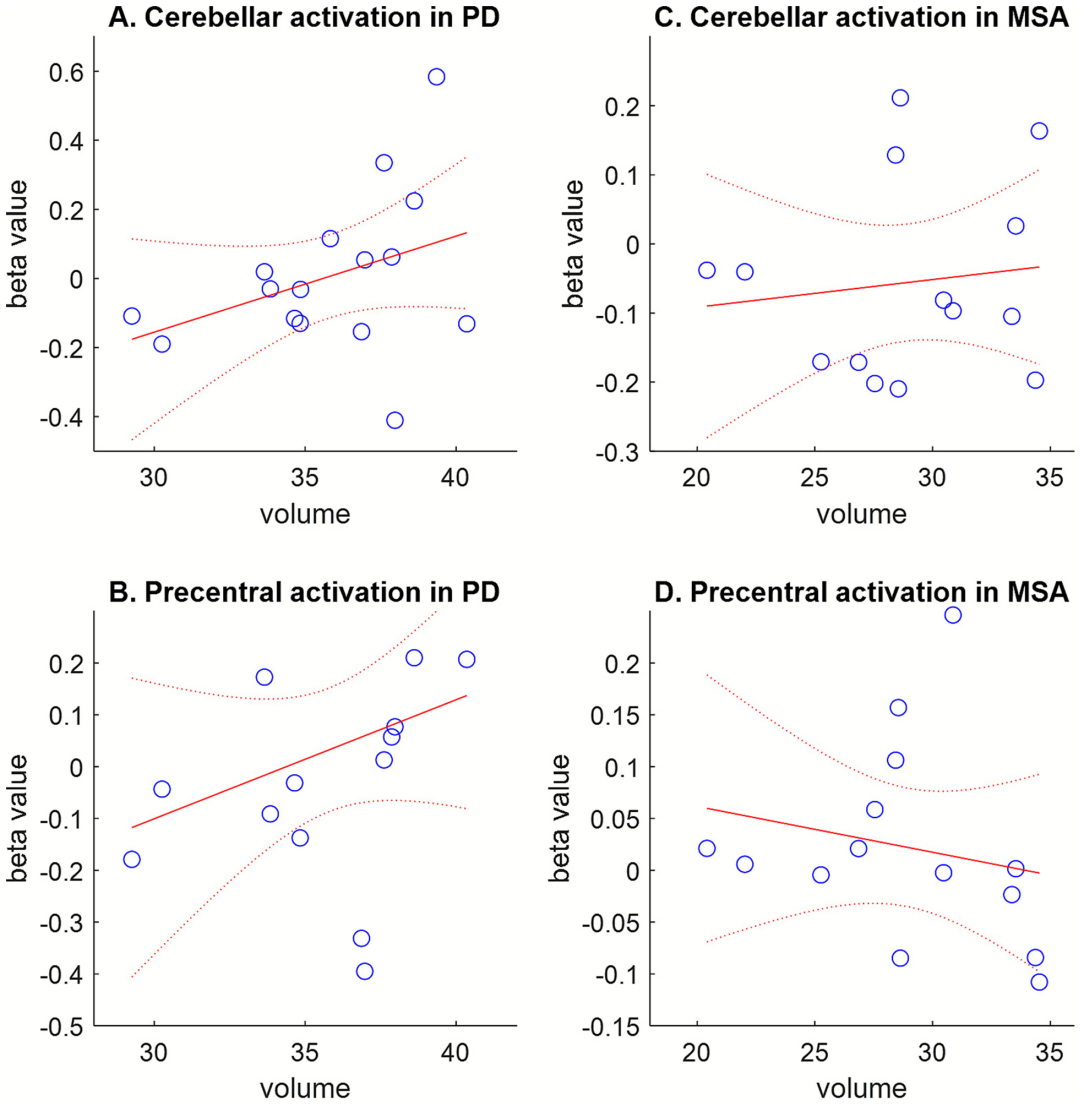
The correlation between activation (beta value) and cerebellar volume. **(A)** Correlation between cerebellar beta value [30, −58, −30] and right cerebellar volume in PD. **(B)** Correlation between precentral beta value [−40, −6, 46] and right cerebellar volume in PD. **(C)** Correlation between cerebellar beta value [30, −58, −30] and right cerebellar volume in MSA. **(D)** Correlation between precentral beta value [−40, −6, 46] and right cerebellar volume in MSA. Cerebellar activation (beta values) showed a positive correlation trend with cerebellar volume in both PD and MSA. A positive correlation between precentral activation and cerebellar volume was also observed in PD, whereas a negative correlation trend was observed in MSA. Solid red line indicates the adjusted linear fit; dotted red lines represent the 95% confidence bounds.

The degree of activation by ABP was defined as the beta value extracted from the SPM results at the voxel of interest. The analysis focused on the right cerebellum because the activation contrast between PD and MSA was localized to the right cerebellum. The Shapiro–Wilk test indicated that the volumes and beta values in both PD and MSA did not deviate from normality. Although the Pearson correlation analysis did not yield statistically significant results, the observed correlation trends may reflect an underlying compensatory mechanism. Specifically, the correlation between cerebellar beta values and right cerebellar volume showed a positive trend in both PD and MSA. In contrast, the correlation between precentral beta values and right cerebellar volume was positive in PD but negative in MSA. Scatter plots and correlations between beta values and cerebellar volume are illustrated in Figure 4, and detailed comparisons are provided in the Supplementary Table.

## 4 Discussion

Our results showed that ABP caused activation in the premotor area in the frontal lobe, and sensory areas in the parietal lobe, striatum/thalamus and cerebellum (Supplementary Figure 1), as also demonstrated in previous studies (Proksch et al., 2020). Under the standard analysis, only partial activation of these regions was observed in the PD and MSA groups when examined separately. Activation in the precentral regions tended to be higher in patients with MSA than in NC (Figures 3C,D), which may reflect compensatory upregulation in response to hypofunction of the cerebellum and basal ganglia in MSA. In contrast, the PD group exhibited greater cerebellar activation than the MSA group (Figures 3A,B); however, cerebellar activation relative to NCs was not significantly increased. Although the NC group is younger, and this contrast may therefore include age-related effects, the primary purpose of the comparison is to demonstrate differences between PD and MSA. If age contributes to the observed effects, the PD > NC and MSA > NC contrasts would be influenced similarly, because the PD and MSA groups are of comparable age. When a more lenient analytical threshold was applied, compensatory activation patterns in motor-related regions became more apparent and were in line with previous findings (Supplementary Figure 2). Specifically, reduced cerebellar engagement in MSA accompanied by increased activation in other motor-associated regions was evident. Nonetheless, these observations should be interpreted as indicative trends rather than definitive effects, given the increased likelihood of false-positive results under the lenient analysis.

**Figure 5:**
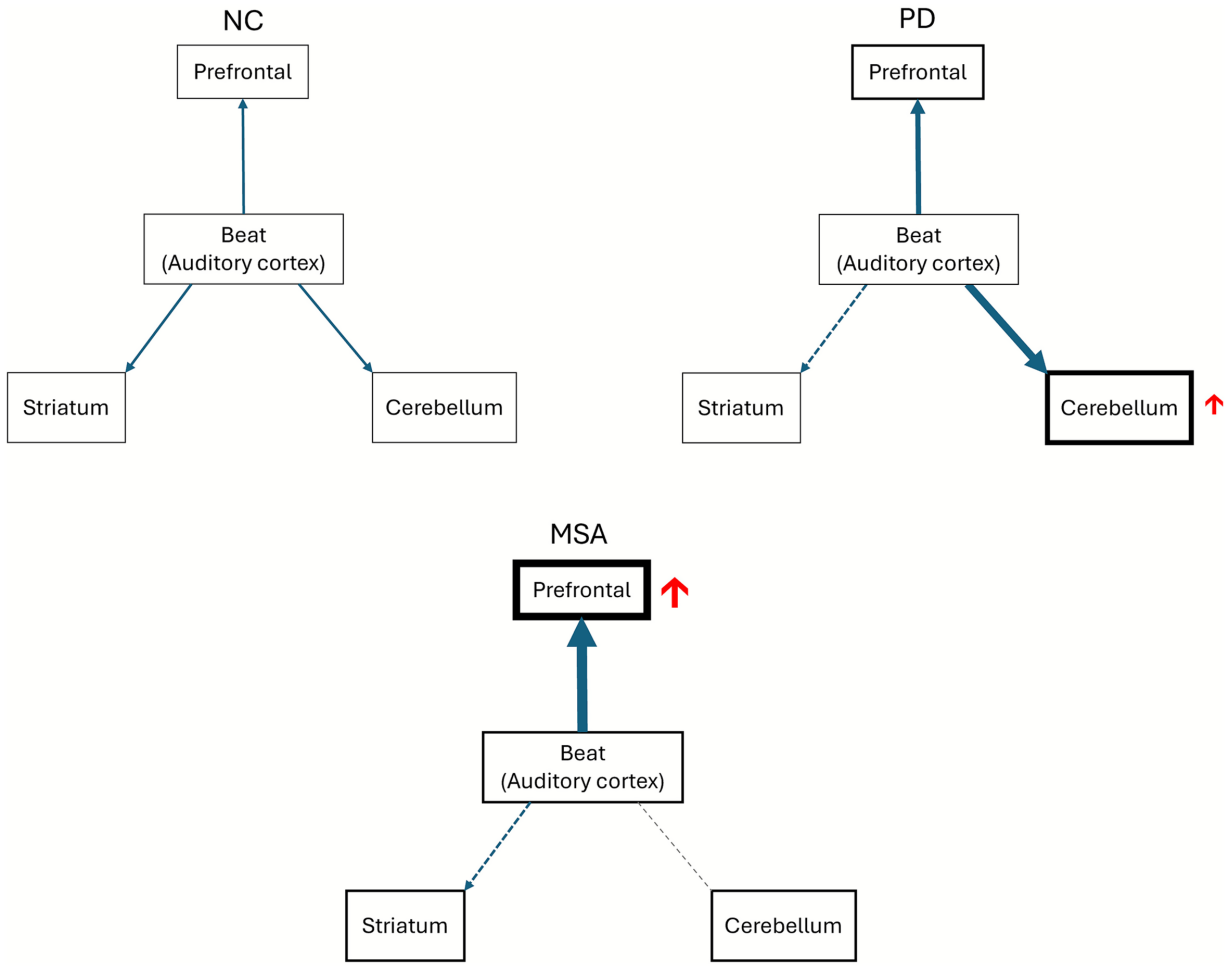
Activation by auditory beat perception, including compensatory responses in the PD and MSA groups. Under normal conditions (NC), auditory cues are processed in the auditory cortex, and beat perception engages the prefrontal cortex, striatum, and cerebellum. In PD, where striatal function is impaired but cerebellar function is relatively preserved, beat perception appears to be compensated by increased cerebellar activation and a slight increase in prefrontal activation. In MSA, characterized by both striatal dysfunction and significant cerebellar impairment (cerebellar atrophy), beat perception relies predominantly on the prefrontal cortex, which shows markedly increased activation.

A plausible explanation for the relatively decreased activation in the cerebellum in the patients with MSA compared to those with PD is the reduction in cerebellar volume. Consequently, the neuron loss may be too severe to elicit compensatory reactions to ABP in the cerebellum. Previous studies comparing differences between PD and MSA in neuroimaging have shown that reductions in cortico-cerebellar connectivity and alterations in cerebellar volume and internal connectivity as elicited by resting-state fMRI were the most striking features to differentiate PD from MSA (Yao et al., 2017; Wang et al., 2019; Baggio et al., 2019). However, the finding of altered cerebellar connectivity was established only in resting-state fMRI but not clearly demonstrated in task-based fMRI. A possible reason is that the tasks did not involve the processes of timing estimation and prediction but only hand grasping, and thus motor processes involving the whole brain were shown (Planetta et al., 2015; Li et al., 2024). In the present study, beyond the resting-state neuroimaging study, task-related hypofunction in the cerebellum was clearly demonstrated and a possible compensatory response was also observed in the PD and MSA groups. In the healthy individuals (the NC group), the basal ganglia, prefrontal region and cerebellum were activated in response to ABP. In the patients with hypofunction in the basal ganglia (the PD group), activation of the prefrontal region and cerebellum was increased to compensate for the insufficient neural activity in the basal ganglia. In the patients with a more advanced condition besides hypofunction of the basal ganglia (the MSA group), cerebellar dysfunction (significant volume loss) was also prominent, and consequently no further compensatory cerebellar responses could be generated. This then led to increased activation in the claustrum as compensation, possibly because the claustrum is thought to be a complex connection hub for both motor and sensory functions. The most striking functional difference between the PD and MSA groups was cerebellar activation (Figures 3A,B), and the prominent difference in regional brain volume changes between the PD and MSA groups was also in the cerebellum. This was partially supported by the observed correlations between compensatory activations in the cerebellum and claustrum and cerebellar volume. Comparisons of neural activation by ABP among the NC, PD and MSA groups are illustrated in Figure 5.

The beat perception process does not involve the same neural circuits and processes as instructed hand grasping actions, and the process requires two main brain areas to estimate the timing. A previous study investigating regular and relative timing showed that it was processed in the basal ganglia, which is thought to be a beat generator in the brain (Teki et al., 2011). In addition, the absolute timing of the interval of each stimulus (or sound) has been shown to be processed in the cerebellum (Teki et al., 2011). As mentioned, the patients in the PD group could not perform beat perception due to dysfunctional basal ganglia, leading to greater activation of the cerebellum (Braunlich et al., 2019). That is, using cerebellar absolute timing perception compensated for the impairment of beat generation in the basal ganglia. In the MSA patients, both basal ganglia and cerebellum were impaired. Consequently, the compensatory mechanism was to increase neuronal recruitment in the premotor area. This then resulted in increased activation in the premotor region as shown in comparisons of the MSA and NC groups. This could also explain why activation in the cerebellum was more prominent in the PD patients compared to the MSA patients, since the function of the cerebellum was less deficient. Because cerebellar activation is crucial for effective RAS gait training in PD patients (Naro et al., 2022), increasing activation of the cerebellum using a rhythm that demands a greater ability to judge absolute timing, such as syncopation, might be feasible. A previous study comparing regular tempo and syncopated rhythm found that cerebellar activation was greater with the syncopated rhythm (Mayville et al., 2002). In addition to gait training, RAS can also be used for adjuvant physiotherapy to improve balance performance (Capato et al., 2020), which may require more cerebellar involvement. Based on the findings of this study, when administering external rhythmic cueing therapy to patients with cerebellar dysfunction, such as those with MSA, a simple regular beat may not be sufficient to effectively activate the cerebellum. This is likely due to compensatory mechanisms that bypass the cerebellum. To enhance cerebellar activation, incorporating rhythmic modulation, rather than relying solely on a regular beat, should be considered as a therapeutic strategy.

There are several limitations to this study. First, the number of cases is relatively small. More cases in each group may have resulted in more robust findings. Second, the participants were only required to listen to the drum beats attentively, which was confirmed by sufficient auditory cortex activation. However, ABP could potentially be enhanced by incorporating more specific tasks, such as judging tempo speed or tapping along with the beats. Finally, the NC group was younger than the parkinsonian groups, and age may have affected comparisons between them. Whether a stronger activation pattern of beat perception is correlated with the responsiveness to RAS in gait performance is also another important issue to be addressed and explored in future studies.

## 5 Conclusion

ABP activates multiple brain regions, and especially the basal ganglia, motor-related regions, and cerebellum. The patients with PD and MSA showed different compensatory processes which were correlated with the functional loss in related brain and cerebellar regions. These findings may provide further insight into the responsiveness to RAS therapy when it is applied to more types of parkinsonian disorders.

## Data Availability

The raw data supporting the conclusions of this article will be made available by the authors, without undue reservation.
