# Supplementary material for: Discriminative compensatory activation during auditory beat perception in Parkinson’s disease and multiple system atrophy
Source: Front Neurosci. 2026 Jan 30;20:1700800. doi: 10.3389/fnins.2026.1700800 (PMC12901494; doi:10.3389/fnins.2026.1700800)
Supplement: Supplementary file 1 [file Data_Sheet_1.docx]

Supplementary Material


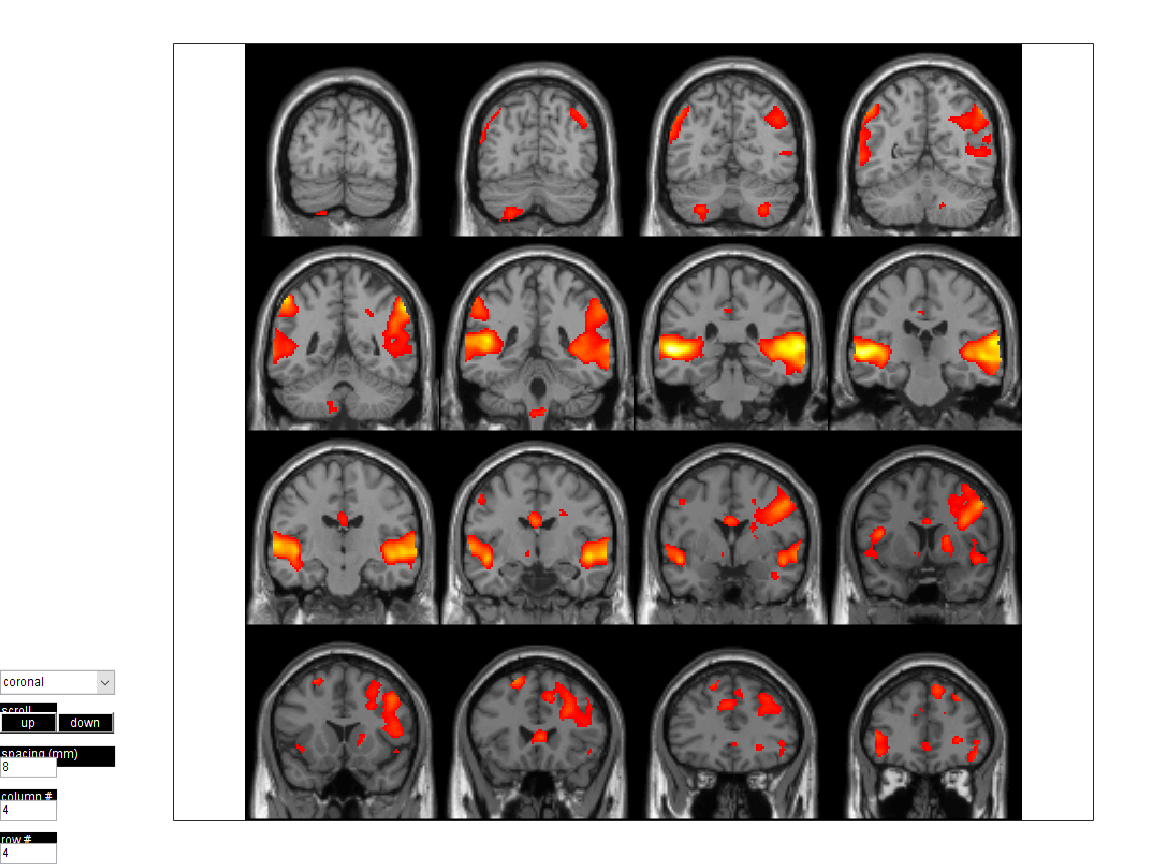


**Supplementary Figure 1.** Overall responses to auditory beat perception of the three groups in coronal (upper part) and axial (lower part) views.


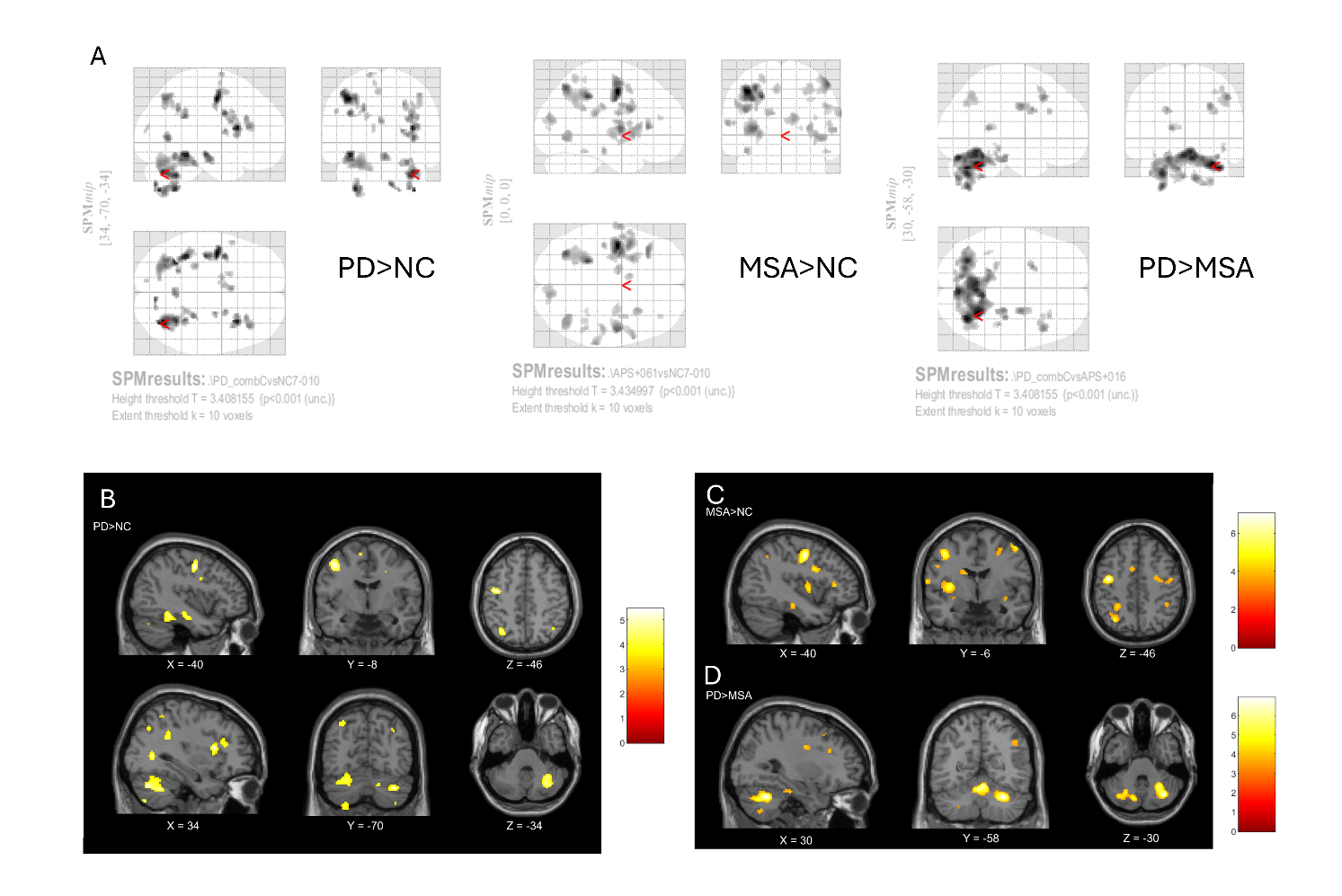
**Supplementary Figure 2.** A lenient threshold using an uncorrected p value < 0.001 with a cluster size of 10. A: The raw activation maps in SPM. The distribution of significant differences can be seen globally. B, C & D: Comparisons between the PD, MSA and NC groups mapping the results onto T1 structural images. B: Contrast between the PD and NC groups. Two main clusters are shown in upper row (precentral area of left frontal lobe, x=-40, y=-8, z=-46, cluster size = 518 voxels) and lower row (Cerebelum_Crus1, right side of the cerebellum, x=-26, y=-72, z=-26, cluster size = 314 voxels). C: Contrast between the MSA and NC groups. The main clusters were in the precentral area of the left frontal lobe (x=-40, y=-6, z=46, cluster size = 317 voxels) and left claustrum (seen in the left part of Fig. 1B). D: Contrast between the PD and MSA groups. The largest cluster was in the cerebellum (Cerebelum_6_R, x=30, y=-58, z=-30, cluster size = 2851 voxels). Another smaller cluster was seen in the right angular gyrus (cluster size of 157 voxels, not shown in the figure). All results are illustrated by a significance level of uncorrected p < 0.001 and a cluster size > 10 voxels. The color bars on the right are the T scores.

**Supplementary Table .** Comparisons of basic characteristics, volumes and correlations among the three groups.

|  | PD  (n=16) | MSA  (n=14) | NC  (n=14) | p-value  (PD vs MSA) | p-value  (PD vs NC) | p-value  (MSA vs NC) |
| --- | --- | --- | --- | --- | --- | --- |
| Basic characteristics | | | | | | |
| Gender (M/F) | 10/6 | 6/8 | 8/6 | 0.464 | 0.529 | 0.706 |
| Age (years) | 56.75 (8.68) | 58.14 (9.46) | 33.57 (7.09) | 0.780 | <0.001 | <0.001 |
| Disease duration (years) | 4.44 (1.21) | 3.71 (0.73) | N/A | 0.061 | N/A | N/A |
| HY stage | 1.81 (056) | 2.86 (0.36) | N/A | <0.001 | N/A | N/A |
| UPDRS-III | 21 (5.82) | 24.71 (10.27) | N/A | 0.280 | N/A | N/A |
| MoCA | 26.63 (2.60) | 25.07 (2.53) | N/A | 0.110 | N/A | N/A |
| Volume (cm^3^) | | | | | | |
| Total gray matter | 459.82 (48.68) | 448.20 (32.92) | 536.24 (44.40) | 0.457 | <0.001 | <0.001 |
| Striatum | 7.25 (1.65) | 6.75 (1.26) | 8.81 (1.13) | 0.367 | 0.006 | 0.001 |
| Cerebellum | 72.35 (6.24) | 57.31 (9.38) | 73.36 (4.93) | 0.006 | 0.632 | <0.001 |
| Activation of BOLD signal at right middle cerebellar cortex [30, -58, -30] | | | | | | |
| Beta value | 0.11 (0.28) | -0.06 (0.14) | -0.19 (0.36) | 0.051 | 0.015 | 0.205 |
| Correlation between right lateral cerebellar volume and beta value (at [30, -58, -30]) | | | | | | |
| Shapiro-Wilk test | 0.277 | 0.095 | 0.005 |  |  |  |
| Rho | 0.366 | 0.124 | 0.130* | PD and MSA groups:  Rho 0.301, p-value 0.107 | | |
| p-value | 0.163 | 0.672 | 0.782* |  |  |  |
| Activation of BOLD signal at left precentral [-40, -6, 46] | | | | | | |
| Beta value | -0.03 (0.23) | -0.01 (0.07) | -0.001 (0.103) | 0.551 | 0.431 | 0.607 |
| Correlation between right lateral cerebellar volume and left precentral beta value (at [-40, -6, 46]) | | | | | | |
| Shapiro-Wilk test | 0.699 | 0.210 | 0.002 |  |  |  |
| Rho | 0.312 | 0.201 | 0.209* | PD and MSA groups:  Rho 0.104 p-value 0.583 | | |
| p-value | 0.240 | 0.491 | 0.479* |  |  |  |

*Spearman correlation coefficients were calculated in NC group, in other two groups the correlation was presented by Pearson correlation coefficients.
